# Surgical Expedients in Emergencies

**Published:** 1883-06

**Authors:** 


### SURGICAL EXPEDIENTS IN EMERGENCIES.

At the recent meeting of the State Medical Society of Pennsylvania, held May 10, 1883, Dr. R. J. Levis (Class of 1848), gave an account of some surgical expedients, in which the skill and readiness of the surgeon are often severely tested. Some of these suggestions, which we find reported in the *Medical News*, were quite ingenious.

In case of an over-distended bladder, where prompt relief is necessary and no catheter is at hand, he had taken a piece of bell-wire, doubled upon itself so as to form a loop, which was readily passed along the urethral canal into the bladder. In a female a rye-straw might be used, its end being rounded with a little sealing-wax, or the stem of a clay-pipe, as crude substitutes for a catheter. In phlebotomy, when a proper lancet is not at hand, an ordinary pocket knife will answer, provided the vein be held in position by transfixing it with a needle after applying the ordinary bandage.

For obstinate epistaxis requiring plugging of the nostril, a piece of sponge, to which a string is fastened, is forced through the meatus to the posterior nares; small pieces of sponge are then to be threaded on this cord and pushed in succession into the passage until it is filled; when the danger of hemorrhage is over, they can be removed by reversing this process. Another good method in an emergency is to take a portion of the intestine of a chicken or other small animal, close one end and pass it through the meatus; water or air may now be forced into the portion in the nostril so as to make equable compression. If it is necessary to plug the posterior nares, a slender gum bougie or a piece of thick catgut ligature may be passed along the floor of the nostril and brought out under the soft palate; a string can then be attached and brought out of the nose in front by withdrawing the bougie; the sponge can then be employed in the usual manner.

In case of bleeding from an intercostal artery from a homicidal wound, he had succeeded in arresting the hemorrhage by introducing the upper part of an ordinary key into the pleural cavity, then turning it at a right angle, and making pressure upon the vessel. After this had been continued for some hours the bleeding ceased.

A very efficient substitute for the Esmarch elastic bandage is a flannel roller cut bias. For dislodging and forcing downward a foreign body in the œsophagus, an ordinary carriage or riding whip, knotted sufficiently far from the end to ensure flexibility, may be used.

Good temporary dressings for fractures may be extemporized by tearing palm-leaf fans into strips; a more permanent fixed dressing can be made by dipping ordinary sand-paper in hot water, and applying it while soft; it adapts itself to the shape of the limb, but becomes sufficiently strong and rigid afterwards; hard dressings can also be made with starch, or eggs and flour.

In moving a patient with fractured thigh, the sound limb may be made into a splint, by fastening the legs together. In treating fractures of the femur, complicated apparatus is not necessary; simple extension by weights is all sufficient, the limb being kept in position by lateral supports or sand-bags. The postural method without splints is to be preferred in all fractures near to joints; fracture of clavicle is best treated by the supine position, with the head slightly elevated.

An ordinary gimlet is an efficient instrument with which to open the mastoid cells in case of abscess and threatening cerebral complication. The carpenter's rasp may sometimes replace the trephine in replacing fragments of bone after fracture of the skull.

A rubber tube may be used instead of a syringe in cases of obstruction of the bowels, the fluid being injected by hydrostatic pressure.

The substitution for belladonna of stramonium, where a mydriatic is needed, and replacing carbolic acid by sulphurous acid, as a disinfectant; and the employment of hot water in place of all other styptics, were also mentioned. Dr. Levis prefers the straight glover's needle in place of the ordinary curved surgical needle for sutures.

